# The ELSO Maastricht Treaty for ECLS Nomenclature: abbreviations for cannulation configuration in extracorporeal life support - a position paper of the Extracorporeal Life Support Organization

**DOI:** 10.1186/s13054-019-2334-8

**Published:** 2019-02-08

**Authors:** Lars Mikael Broman, Fabio Silvio Taccone, Roberto Lorusso, Maximilian Valentin Malfertheiner, Federico Pappalardo, Matteo Di Nardo, Mirko Belliato, Melania M. Bembea, Ryan P. Barbaro, Rodrigo Diaz, Lorenzo Grazioli, Vincent Pellegrino, Malaika H. Mendonca, Daniel Brodie, Eddy Fan, Robert H. Bartlett, Michael M. McMullan, Steven A. Conrad

**Affiliations:** 1 ECMO Centre Karolinska, Department of Pediatric Perioperative Medicine and Intensive Care, Karolinska University Hospital, 171 76 Stockholm, Sweden; 2 Department of Physiology and Pharmacology, Karolinska Institutet, Stockholm, Sweden; 3 Department of Intensive Care, Hôpital Erasme, Université Libre de Bruxelles, ULB, Brussels, Belgium; 4 Cardio-Thoracic Surgery Department - Heart & Vascular Centre, Maastricht University Medical Hospital, Cardiovascular Research Institute Maastricht (CARIM), Maastricht, Netherlands; 5 Department of Internal Medicine II, Cardiology and Pneumology, University Medical Center Regensburg, Regensburg, Germany; 6 Department of Cardiothoracic Anesthesia and Intensive Care, Advanced Heart Failure and Mechanical Circulatory Support Program, San Raffaele Hospital, Vita Salute University, Milan, Italy; 7 Pediatric Intensive Care Unit, Children’s Hospital Bambino Gesù, IRCCS, Rome, Italy; 8 U.O.C. Anestesia e Rianimazione 1, Fondazione IRCCS Policlinico San Matteo, Pavia, Italy; 9 Department of Anesthesiology and Critical Care Medicine, Johns Hopkins University School of Medicine, Baltimore, MD USA; 10 Department of Pediatrics, University of Michigan, Ann Arbor, MI USA; 11 Clinica las Condes S.A, Santiago, Chile; 12 Ospedale Papa Giovanni XXIII, Bergamo, Italy; 13 Alfred Hospital, Melbourne, Victoria Australia; 14 Inselspital, Children’s University Hospital, Bern, Switzerland; 15 Department of Medicine, Columbia University College of Physicians and Surgeons/New York-Presbyterian Hospital, New York, NY USA; 16 Interdepartmental Division of Critical Care Medicine, University of Toronto, Toronto, Ontario Canada; 17 Department of Surgery, University of Michigan, Ann Arbor, MI USA; 18 Seattle Children’s Hospital, Seattle, WA USA; 19 Louisiana State University Health Sciences Center, Shreveport, LA USA; 20 European ECMO Advisory board, Pavia, Italy

**Keywords:** Extracorporeal life support, Membrane oxygenation, Nomenclature, Abbreviation, Configuration, Cannula, ELSO

## Abstract

**Background:**

The Extracorporeal Life Support Organization (ELSO) Maastricht Treaty for Nomenclature in Extracorporeal Life Support (ECLS) established consensus nomenclature and abbreviations for ECLS to ensure accurate, concise communication.

**Methods:**

We build on this consensus nomenclature by layering a framework of precise and efficient abbreviations for cannula configuration that describe flow direction, number of cannulae used, any additional ECLS-related catheters, and cannulation sites. This work is a consensus of international representatives of the ELSO, including those from the North American, Latin American, European, South and West Asian, and Asian-Pacific chapters of ELSO.

**Results:**

The classification increases in descriptive capability by introducing a third (cannula tip position) and fourth (cannula dimension) level to those provided in the previous consensus on ECLS cannulation configuration nomenclature. This expansion offers the simplest level needed to convey cannulation information yet allows for more details when required.

**Conclusions:**

A complete nomenclature for ECLS cannulation configurations accommodating future revisions was developed to facilitate ability to compare practices and results, to promote efficient communication, and to improve quality of registry data.

**Electronic supplementary material:**

The online version of this article (10.1186/s13054-019-2334-8) contains supplementary material, which is available to authorized users.

## Background

The Extracorporeal Life Support Organization (ELSO) Maastricht Treaty for Nomenclature in Extracorporeal Life Support (ECLS) was recently published [1] as a consensus among several stakeholders for the nomenclature and abbreviations in ECLS to ensure accurate, concise communication. Through this effort, a flexible and modifiable format was established that describes current ECLS cannulation configurations but is also adaptable for anticipated future revisions.

ECLS is defined as a set of therapies that focus on oxygenation, carbon dioxide removal, cardiac support, or a combination thereof [1]. Cardiopulmonary bypass for cardiothoracic or vascular surgery is excluded from this definition. Extracorporeal membrane oxygenation (ECMO) is one ECLS entity used for temporary support of patients with respiratory and/or cardiac failure. Recent research and development have transformed ECLS, which is now being delivered at increasing frequency to patients around the world [2–7]. The global diversity of approaches to temporary support with ECLS necessitated the consistent nomenclature that was recently released by the ELSO (Ann Arbor, MI, USA) [1] to benefit clinical and research practices. One of the first steps in creating the nomenclature was to separate *mode* from *cannula configuration.* The mode comprehensively describes the type of organ support delivered, whereas the configuration describes where the cannulae are implanted and where the tip is positioned.

The basic ECLS modes—venovenous (VV) and venoarterial (VA)—have evolved considerably over the years, with additional indications for use, including strategies that have rather different intensions for support (i.e., bridge to recovery, bridge to transplant or surgery, bridge to decision, and bridge to destination). As a result, clinicians started to employ new cannulae and new cannula configurations for ECLS management, either from the start or during support based on the clinical conditions [8–10]. For example, the use of an additional (“third”) cannula was progressively implemented to increase the capacity for venous drainage or to improve the systemic oxygenation in VA ECMO patients (e.g., by adding an additional venous return cannula—the hybrid mode VVA). Without a formalized approach to nomenclature, inconsistencies arose between studies and settings, between modes and configurations. Hence, in one center, “VVA” could represent VA ECMO with dual return cannulae, one in an artery and one in a vein, while in another center it might represent dual venous draining points in a VA ECMO. Thus, “VVA” or “VAV” may be used in the same center for VA ECMO with dual return cannulae, depending on whether the initial mode was VV or VA [10]. Separately, smaller catheters may be placed to promote distal perfusion of a cannulated lower extremity, e.g., on the arterial side [11, 12], or to improve drainage or release congestion of an extremity, the heart, or head (cephalad catheter) [12–16]. Abbreviating these cannulae adds further opportunity for confusion.

The aim of this work is to describe the stepwise derivation of the nomenclature for the creation of straightforward abbreviations for cannulation configurations in ECLS.

## Methods

This document represents a consensus on abbreviations for the ELSO nomenclature for peripheral and central cannulation configurations in ECLS. The contributors represent multiple specialties that perform ECLS, including cardiothoracic surgery, pediatric surgery, surgical intensive care, anesthesiology, cardiology, pulmonary medicine, medical intensive care, pediatric intensive care, neonatology, and emergency medicine. The contributors also represent the international chapters of the ELSO, including the North American, Latin American, European, South and West Asian, and Asian-Pacific, conveying a global perspective on ECLS. The initiative for this detailed classification was formalized in Maastricht, The Netherlands, at the 6th Euro-ELSO annual meeting in 2017, as the Nomenclature Task Force assembled by ELSO decided to publish a consensus statement on terms, abbreviations, and synonyms, the definitions of which were implemented by ELSO earlier this year [1]. In that document, a brief presentation of the nomenclature and taxonomy for ECLS cannulation was provided.

The current document is the expanded detailed description of the ELSO nomenclature for peripheral and central ECLS. The classification introduces third and fourth description levels to the two levels provided in our previous work [1]. This expansion offers the simplest level needed to convey cannulation information yet allows for more details when required.

The full ECLS cannulation nomenclature will be structured in these four levels:

1. Configuration
2. Cannulation site
3. Tip position
4. Cannula dimension

The manuscript will explain the four levels of the nomenclature in detail. The full nomenclature will allow practitioners to use standardized abbreviations for all ECLS modalities. Its goal is to establish consistency for clinical and research descriptions.

## Results

### Definitions of cannulation

**Fig. 1:**
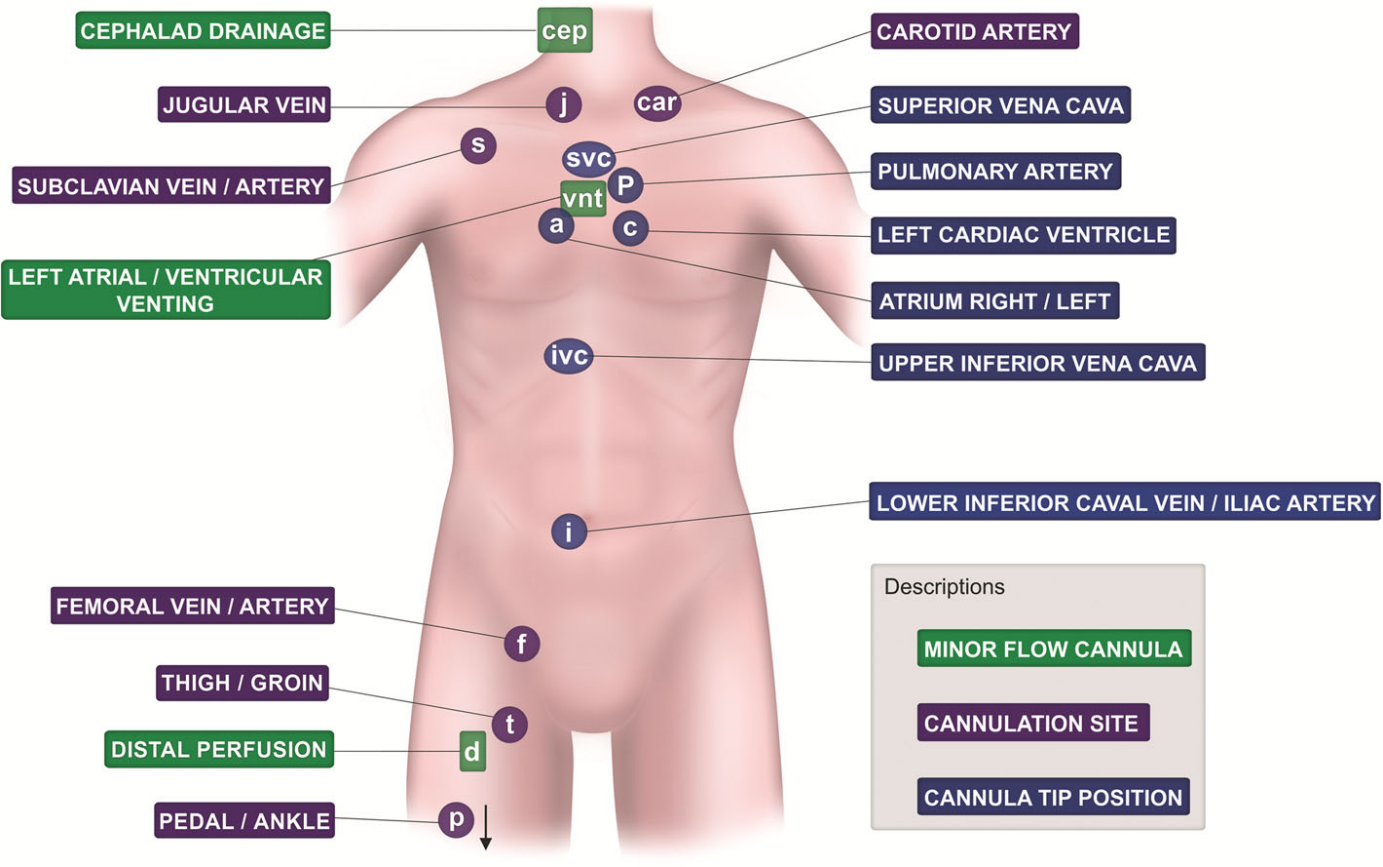
Anatomical locations for cannulation and tip positioning. All potential peripheral cannulation sites of venous/drainage or arterial/return can be easily identified with different letters (see text for further details). Cannula tip positions are indicated by blue circles

*Peripheral cannulation* is defined as a cannulation in which the vessel puncture, venotomy or arteriotomy, is performed on a vascular structure(s) outside the thoracic or abdominal cavity (Fig. 1) [1]. Such cannulation may be performed with the Seldinger technique, a cut-down procedure, or a combination of both (semi-Seldinger technique).

*Central cannulation* is defined as any cannulation on cardiac structures, intrathoracic aorta, or pulmonary artery. These procedures require sternotomy or thoracotomy [1], although percutaneous access has been anecdotally described with already available or dedicated cannulae and may, therefore [17], be used more frequently in the future.

### The ELSO classification for peripheral cannulation configurations

#### Flow direction

**Fig. 2:**
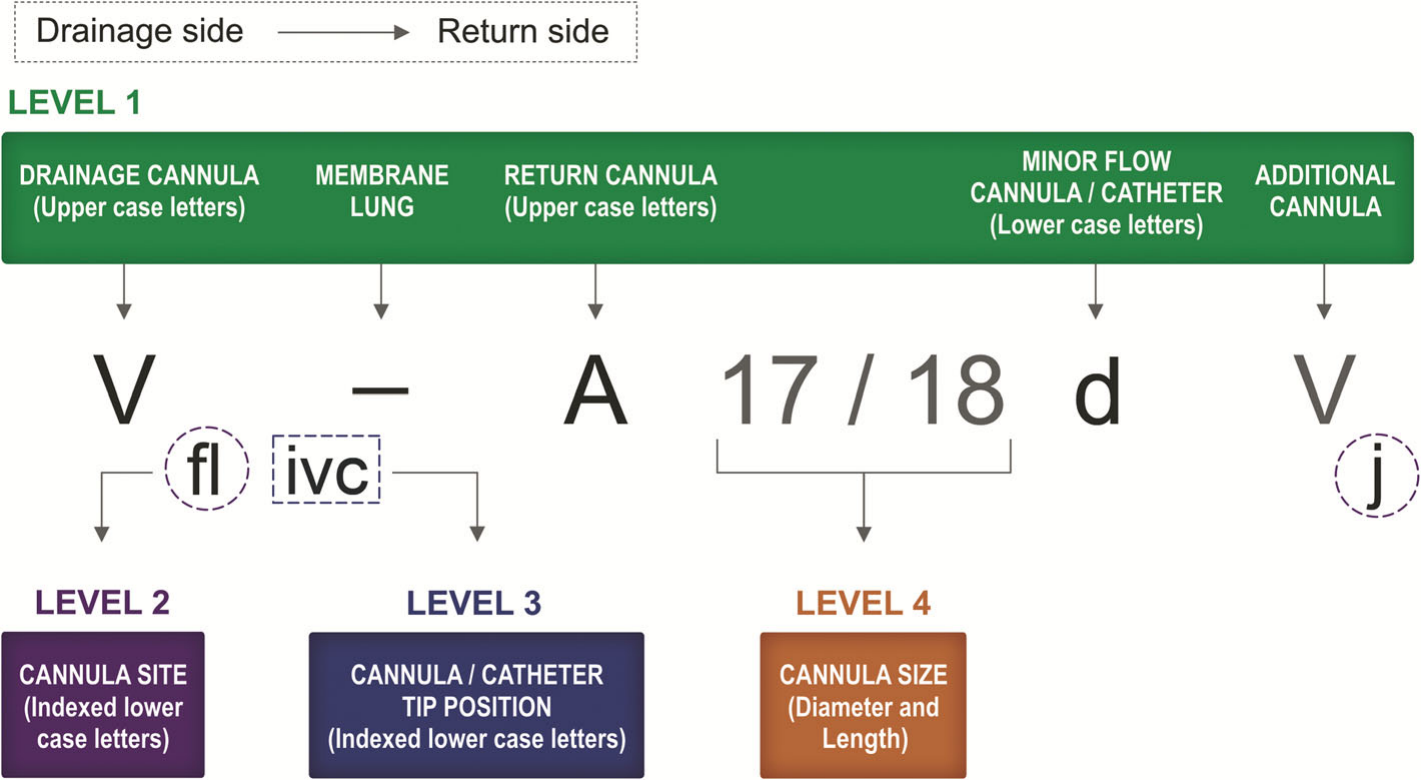
Different levels of the nomenclature. The nomenclature levels are presented in order from Level 1 to Level 4. Abbreviations may be used according to each center’s praxis from Level 1 or more detailed. Levels may be combined according to the user’s preference, e.g., Levels 2 and 4. The drainage cannula is placed on the left of the hyphen (“**-**”). The return cannula is placed on the right side. More precise positions (that do not directly relate to a cannula or catheter) are given as indexes, e.g., femoral (f), left (l) or right (r), atrium (a). For the complete lists of abbreviations, see Table 2 and Additional file 1

The basic proposal for ECLS configuration is to describe the direction of blood flow from left to right, with the drainage side on the left and the point at which oxygenated blood is returned to the patient on the right (Fig. 2).

#### Level 1: Cannula hierarchy

All cannulae contributing to a *major* drainage or return flow are written in *uppercase* letters. All cannulae or catheters with a *minor* flow for unloading of a specific anatomical location or promoting perfusion are written in *lowercase* letters *after* the major flow cannula to which side it belongs (Additional file 1 and Additional file 2 section A). Additional file 1 shows the whole nomenclature and may support the text that follows.

To increase consistency, a hyphen is used to illustrate the relative position of the extracorporeal membrane lung (ML) in the configuration described. Thus, for single-lumen cannula applications, the drainage cannula will always be to the left of the hyphen, and the return cannula will be to the right of the hyphen (Fig. 2). As such, a two-letter mode cannot be misinterpreted and will be kept designated (“V-V” or “V-A” cannulation), where V indicates venous, A indicates arterial, and - indicates the ML. A second ML used in parallel to another will be indicated as “V**=**V”, and rarely, if the second ML is put in series, the configuration would be indicated as “V**+**A”. To allow for pure peripheral cardiac support, i.e., no ML in circuit, “x” replaces the ML (VxA).

This nomenclature would also be applied to pumpless ECLS (e.g., “A-V” for AV ECCO_2_R) [18, 19]. In a pumpless configuration (peripheral or central), “pl” is placed directly before the drainage cannula in parentheses, (pl)A-V. If a V-V configuration uses a double-lumen cannula (DLC), the abbreviation “dl” should be used in parentheses before the first “V”, (dl)V-V. For an even greater degree of detail, “dl” could be exchanged for “bc” or “ca” to indicate the use of a bi-caval or cavo-atrial DLC, respectively.

When a third (and sometimes fourth) cannula is used, that additional uppercase letter is placed accordingly to the outer side of the already existing cannulae, which constitute the core. As an example, if a second venous drainage cannula is added, that “V” will be put to the left of the hyphen and outside of the first drainage cannula: “VV-A”. If during V-A, an additional re-entry cannula is placed in any vein to improve systemic oxygenation in parallel to the initial arterial cannula, that “V” is added to the outer right side because it represents post-ML flow (return): “V-AV”, If an arterial cannula is added to a “V-V” configuration, that would be expressed as “V-VA”. Thus, over a course of an ECMO run, the configuration order will in part describe the patient’s temporal needs for support (i.e., chronology). If a VVA mode configuration is implanted in one instance and the major concern is cardiac support, V-AV should underline this fact. If the reason is unknown, the recommendation is to keep in line with the VVA mode, i.e., V-VA. The nomenclature does not allow for discrimination of the respective VV and VA proportional support, and in regard to physiology, V-VA is the same as V-AV. However, the hyphen should be included in the text, and for oral communication, it is necessary to say, especially concerning VV-A, “Vee-and-Vee-to-A”, and for V-VA, “Vee-to-Vee-and-A”.

In the rare case of a patient who has dual venous draining points followed by oxygenated blood pumped back to both the venous and arterial sides, the correct abbreviation would be “VV-VA” (or VV-AV). If VV ECMO is running with three cannulae (e.g., two venous drainage cannulae), the abbreviation would be “VV-V”.

In the case of V-A configuration with a femoral arterial cannula, a distal perfusion cannula may be placed to enhance perfusion of the cannulated leg. Such a *minor flow* catheter will be denoted with a lowercase “d”. As an example, a venoarterial ECMO configuration with distal reperfusion would be indicated as “V-Ad”. A lowercase “d” always follows directly after the uppercase “A”—the cannula it supports. For the special circumstance in which a patient receives a recently developed single-lumen cannula for femoral arterial return, designed with an in situ port for limb reperfusion (i.e., a punched hole on the cannula), the “A” and “d” will be underscored (“Ad”). Because the cannula is one single-lumen device, the letters are not separated. Thus, there is no risk for misunderstanding at any level of hierarchy (V-Ad vs. V-Ad). Jugular venous bulb catheters, which are known as “cephalad” cannulae and used typically in neonates or children as an adjunct to increase venous drainage, augment circuit flow, and decompress cerebral venous pressure during ECMO, will be indicated by a lowercase “cep”: (Vcep-V). The identification for atrial or ventricular venting is “vnt”, as exemplified by Vvnt-A. The lowercase letter is always placed directly after the major cannula to which it interrelates.

If a DLC is being used, and an additional arterial cannula must be added for circulatory support (VVA mode), the abbreviation would be “(dl)V-VA”. If the DLC limbs are unified (Y-connector) for drainage and an arterial cannula added (VA mode), the configuration would be (dl)VV-A. The novel hooked design DLC for right ventricular support would be “(dl)V-P”. The curved cannula passes via the superior vena cava, right atrium (RA, its drainage site), right ventricle, and out to the pulmonary artery. Finally, if a single venous drainage cannula is used, and the re-entry cannula is inserted through the jugular or subclavian vein up to the pulmonary artery to support the right ventricle, the abbreviation would be “V-P”, as shown in Table 1.

**Table 1:** Nomenclature for configuration of peripheral cannulation in extracorporeal life support

Modes		VV (ECMO)		VA (ECMO)	VVA (ECMO)			AV (ECCO_2_R)
Flow direction, from ➔ ML➔ to		V-V, VV-V	V-V	V-A, VV-A	V-VA	V-VA	V-AV	A-V
Configuration		Single-lumen cannulae	Dual-lumen cannula	Single-lumen cannulae	Single-lumen cannulae from V-V	Dual-lumen cannula from V-V	Single-lumen cannulae from V-A	Single-lumen cannulae
Level 1: Hierarchy	Upper case = major flow cannula Lower case = minor flow cannula	Vcep-V	(dl)V-V, (ca)V-V, (bc)Vcep-V	V-Ad, (dl)VV-Ad	Vv-VAd	(dl)V-VA	V-AdV	(pl)A-V
Level 2: Cannulation site	Indexed	V_f_-V_j_	(dl)V_f_-V, (ca)V_j_cep-V	V_j_-A_f_, V_j_V_f_-A_f_d, V_j_-A_car_	V_f_-V_j_A_f_	(bc)V_j_-VA_f_d	V_j_-A_f_V_f_	(pl)A_fl_-V_fr_
Level 3: Tip position	Indexed	V_ivc_-V_a_, V_ja_cep-V_f_	V_fsvc_-V_f_	V_ja_-A_fli_d_p_vnt_al,_ V_ja_-A_srg_, V_j_-A_i_	V_f_-V_fivc_A_f_, V_f_-V_j_A_i_	(ca)V_j_-VA_slc_	V_ja_v_c_-A_fr_d_p_V_fr_	(pl)A_fri_-V_fli_
Level 4: Cannula dimension	OD/L, L is never given unless OD first	V21/50-V17, V21_f_-V17_fivc_ V23/25_a_-V17_f_	(dl31)V-V, (ca32)V_j_cep-V	V25/25-A17/18, V29_fa_-A_f_d_t_ V25_flsvc_-A_fl_19d_p_	V25/38_j_-V_f_ A19/18_f_d_t_	(dl23)V-VA_f_	V25/25_ja_v_a_-A21_fr_d_p_V17/50_fr_	(pl)A15/17_fr_-V15_fl_

*Abbreviations*: (−), membrane lung, *bc* bi-caval dual-lumen cannula, *ca* cavo-atrial dual-lumen cannula, *dl* dual-lumen cannula, *ECMO* extracorporeal membrane oxygenation, *ECCO2R* extracorporeal carbon dioxide removal, *VA V-A* venoarterial, *VV V-V* venovenous, *VVA* venovenoarterial

Level 1: A, arterial; cep, cephalad drainage; d, distal perfusion of cannulated leg; P, pulmonary artery; pl, pumpless; V, venous; vnt, cardiac venting

Level 2: car, carotid; f, femoral; g, chimney graft; j, jugular; l, left; r, right; s, subclavian

Level 3: a, atrium (V_a_, “a” index for venous cannula tip in right atrium; vnt_al_, “al”, (“ar”) index for venting catheter in left (right) atrium); c, left cardiac ventricle (vnt_c_, “c” index for venting catheter position); i, iliac vein/lower inferior vena cava, or iliac artery; ivc, hepatic vein level in inferior vena cava; l, left; p, pedal (ankle), (d_p_, “p” indicate distal (pedal) retrograde perfusion at ankle of cannulated leg); r, right; svc, superior vena cava; t, thigh (groin), (d_t_, “t” denotes distal perfusion with cannula placed in femoral cannulation site)

Level 4: L, cannula length in centimeters; OD, cannula outer diameter in French (1 Fr = 1/3 mm)

#### Level 2: Cannulation site

Level 2 describes which vessel is punctured/cannulated. This information is supplied by using indexes (subscript letters), as in the International Union of Pure and Applied Chemistry (IUPAC) terminology for organic chemistry [20] or the tumor, nodule, metastasis (TNM) classification for malignant tumors [21]. For communication software that does not support subscripting, the indexed letters should be placed in parenthesis directly after the related cannula. For the DLC abbreviation, the lowercase (dl) is always placed before the V-V and cannot be misunderstood. The following *Level 2* examples with indexes thus explain the site for ECMO cannulation (see Additional file 1). V-V ECMO in femoro-femoral flow direction would be represented as “V_f_-V_f_”, [or V(f)-V(f)]. “V_j_-V_f_” would thus be the alternative jugulo-femoral mode of V-V ECMO and “V_f_-V_j_” a femoro-jugular VV ECMO. For V-A configurations, “V_j_-A_f_” would be the jugulo-femoral V-A ECMO, “V_f_-A_f_” would be the femoro-femoral V-A ECMO, and “V_f_-A_f_V_j_” would be V-AV ECMO in which the patient has a single drainage cannula via a femoral vein and dual return cannulae, one via a femoral artery and the other via the jugular vein. The nomenclature also allows description of the cannulated side. Left is denoted as “l,” and right “r”. The side-specific indexed letter is placed after the vessel, V_f**r**_-A_f**r**_. For DLC, it is presumed that the site of cannulation and placement of tip are algorithm-congruent and so should not be reported. However, if new DLC cannula designs appear in the future, the use of (dl)V_j_-V, or (dl)V_fr_-V (via right femoral vein), etc. may be used to specify cannulation site in accordance with this nomenclature. If the index is placed after the second “-VV”, it would indicate the rare case of an extra venous return cannula, (dl)V_j_-VV_f_. A DLC may also be used for V-VA ECMO with an additional arterial infusion cannula: (dl)V_j_-VA_fr_d. The distal perfusion cannula is placed at the same side as the right femoral return cannula (A); thus, side subscript after “d” is not necessary.

#### Level 3: Cannula tip position

To use the index system to its full extent, the point of the cannula tip should also be indexed. This information will increase the readers’ ability to extrapolate the net point for drainage—or point of reintroduction of oxygenated blood—as this knowledge is crucial to understanding both recirculation fraction in VV ECMO [9] and the potential to develop differential hypoxemia in VA ECMO [22–24]. Venovenous ECMO drainage via a femoral vein with the tip placed in the upper inferior vena cava at the level of the hepatic vein (designated “ivc”) and return of the oxygenated blood (via the right jugular vein) in the right atrium (a) would be noted as “V_ivc_-V_a_”. A “V_j_-V_f_” ECMO would be represented as “V_a_-V_f_” ECMO (atrio-femoral flow mode). In recent years, different modes of cannulation that promote physiotherapy and mobility during bridge to transplant have been introduced [25, 26]. These modes would be regarded as part of an *extended definition.* The configuration in which a chimney graft is adapted to the right subclavian artery [21] would be V-A_srg_, where “s” is subclavian artery, “r” is right side, and “g” denotes graft (see Additional file 1). A case of a femoro-femoral venoarterial ECMO with distal perfusion in the groin (thigh) would be indicated as “V_f_-A_f_d_t_”. A pedal distal perfusion catheter will be indicated as “d_p_”. The abbreviation with indexes for left atrial (indexed “a” for tip position) venting is “vnt_al_”, right “vnt_ar_”, and left heart chamber (“c”) vent is “vnt_c_”. When “a” or “c” occur to specify a venting cannula, that letter can never mean anything else. A complete list of all potential sites of peripheral cannulation is shown in Fig. 1.

#### Level 4: Cannula dimensions

**Fig. 3:**
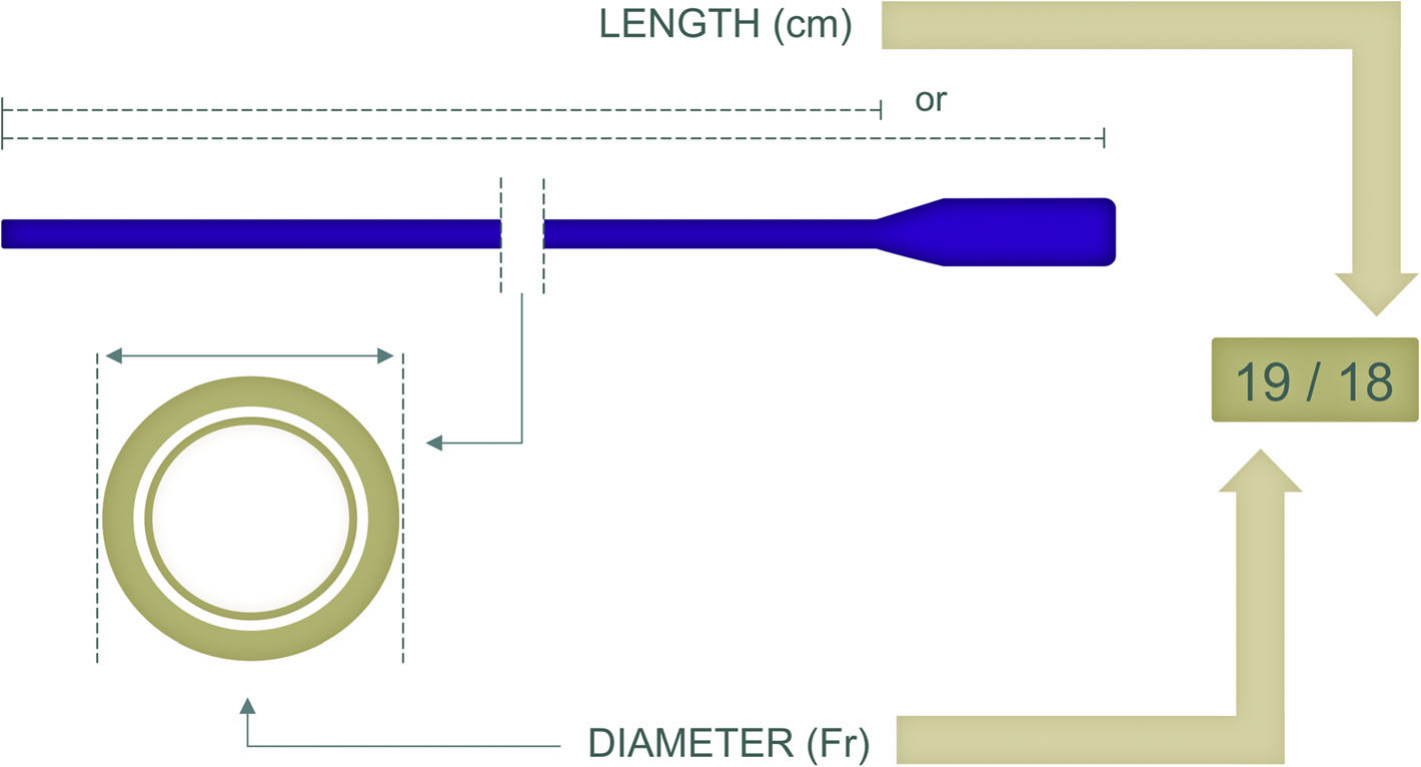
Cannula length and diameter. Length and diameter of each cannula are reported as shown in this example. The diameter printed refers to the outer cannula diameter

Finally, to specify the cannula dimension, the cannula size in French units (1 Fr = 1/3 mm outer diameter) should be added after the respective letter; V17-V15 indicates venovenous mode with a 17-Fr drainage and 15-Fr return cannula. Additionally, cannula length in centimeters could be added only if diameter is reported using the slash (“/”) symbol as: V18/20 to indicate a size of 18 Fr and a length of 20 cm (Fig. 3).

It should be noted that current manufacturers have no uniform definition for “cannula length”. The insertion length (i.e., the distance from tip to where the cannula diameter starts to increase) is the measure of length that is the most appropriate concerning cannula flow physics and placement in the patient.

The rules applying to the complete detailed description of the nomenclature are shown in Additional file 3.

### The ELSO classification for central cannulation

In central cannulation, the rules and hierarchy from peripheral configurations apply but will be more direct. All abbreviations for anatomical entities into which a major flow cannula is placed are expressed in two non-separable capital letters (Table 2). The site and tip position will in most cases be the same. Any minor flow *catheter* (i.e., venting cannula) is expressed as a lowercase letter. Central cannulation ECMO may be applied via conventional sternotomy, or when a membrane lung is incorporated, via any type of ventricular assist device circuit. If an ML is placed in the circuit of an assist device, the prefix “oxy” is added before the device (e.g., oxyLVAD). Examples are shown in Additional file 2, section B.

**Table 2:** Abbreviations used for extracorporeal life support central cannulations for cardiac and/or gas exchange support^a^

Anatomical primary (not indexed)	Abbreviation	Comment
Cannula placed in right atrium	RA	
Cannula placed in right ventricle	RV	
Cannula placed in pulmonary artery	PA	Direct cannulation
Cannula placed in left atrium	LA	
Cannula placed in left ventricle	LV	
Cannula placed in ascending aorta	AO	
Cannula placed in the innominate artery	IA	Chimney graft may be assumed
Atrial venting	vnt_al_ vnt_ar_	Atrial drainage catheter (l, left; r, right)
Left chamber/ventricular vent	vnt_c_	Left chamber/ventricle drainage catheter
Left atrium vent	vnt_ts_	Trans-septal left atrium drainage catheter
Pumpless driven extracorporeal flow	pl	“pl” placed first in abbreviation
Transvalvular left ventricular support	TVLS	Transvalvular axial pump
Transvalvular right ventricular support	TVRS	Transvalvular axial pump
Left ventricular assist device	LVAD	If a membrane lung is put into the circuit: oxyLVAD
Right ventricular assist device	RVAD	If a membrane lung is put into the circuit: oxyRVAD
Intra-aortic balloon pump	IABP	

^a^Systems may be pump-driven or pumpless

### Combination of central and peripheral applications

Central and peripheral configuration abbreviations may be combined. Thus, abbreviations for cardiac venting are shown in Table 2, although they are also used for peripheral ECMO setups. In combinations of central and peripheral ECLS, the drainage to return is still from left to right; thus, either central or peripheral may be written first. The division between peripheral and central is made by a slash “**/**”. For bridge to transplant, some centers use a combination of peripheral venous drainage and central cannulation return via a chimney graft attached to the innominate artery [27]. The abbreviation V_a_-/IA_g_ denotes drainage from the right atrium, ML, and return flow to the innominate artery (IA) via such graft (g). If the hyphen is omitted (V_a_/IA_g_), only cardiac support is provided. Note that the “x” used for peripheral pure cardiac support to mark the absent ML is not needed here because the *slash* represents the interface. More examples are shown in Additional file 2, section C.

### Parallel independent devices

If two devices for any application are used in parallel, their abbreviations are separated by a back slash “\”. Thus, in central cannulation configurations, two supportive devices could be run independently in parallel. The nomenclature allows for this also in peripheral cannulation configurations. A few centers worldwide may adapt two separate (peripheral and/or central cannulation) ECMO circuits to septic patients to allow high blood flows in the resuscitative phase (personal communication: Warwick Butt, Royal Children’s Hospital, Melbourne, Australia). To exemplify, V_fl_-A_fr_d_t_\V_ja_-A_sg_ is an abbreviation for two separate ECMO circuits, where the first drains via the left femoral vein with return flow via the right femoral artery with a distal perfusion cannula in the right groin. The second circuit drains via a jugular cannula with the tip in the right atrium and return flow via either subclavian artery where the return cannula is put into a chimney graft.

### Chronology

If devices are applied at different time points, the first circuit applied should be to the left. If both are implanted during the same procedure, the one regarded as the most important (if one is) should be put first. The same accounts for simultaneous implantation of all cannulae for a VVA mode peripheral configuration, or any other setting that might carry an inherent time sequence.

## Discussion

This classification system is presented with the goal of providing a more uniform and informative nomenclature for ECLS cannulation configurations. The system is intended to allow a quick and comprehensive overview of the ECLS techniques and cannulae, with minimal risk of misunderstanding. This classification includes (1) the ECLS mode, (2) the flow direction, (3) cannulation sites, (4) additional ECLS-related catheters, (5) the flow distribution (if > 2 cannulae), (6) cannula tip position, and (7) cannula dimensions. Simpler combinations of central and peripheral configurations can also be described with this system.

### Special consideration

In a context where differential hypoxemia on VA ECMO (North-South syndrome) may occur, it should be noted that the nomenclature may contain information concerning the cannula tip positions. However, that information does not necessarily reveal the actual drainage point for deoxygenated blood [24]. In the end, clinicians may choose to adopt this system to the level that provides the depth of information they require. The level may vary depending on the setting or nature of the communication. Irrespective of the depth of classification employed, the main goal of this new system is to provide uniformity to the nomenclature.

### Future applications

Future revisions may be needed to cover new technologies and alternate cannulation configurations that might be developed for implantable membrane lungs, such as for carbon dioxide removal (“intra-corporeal” or “implant”, ICO_2_R) or oxygenation (i.e., implantable membrane oxygenation, IMO or ICMO). To facilitate and propagate the use of this complex nomenclature, we are developing a mobile device application that will allow users to work with the nomenclature without needing to refer back to the manuscript.

## Conclusions

This nomenclature could be used in its most simple form, with only ECLS mode and cannula site(s), or with a complete approach, as dictated by local practice in different ECMO centers. The benefits of using this uniform nomenclature include the ability to compare practices, and results, all over the world; more efficient communication of ECMO configurations and additional interventions for flow or oxygenation; and improved quality of registry data, which are used largely to provide epidemiologic information and outcome data for ECMO patients. One limitation of this approach is the inability to describe flow properties according to the design of the cannula in use. We also acknowledge that when it is used to its fullest extent, the terminology is highly complex. However, the flexibility of this nomenclature allows for uniformity of terminology even when only the initial lower levels are used.

## Additional files

Additional file 1: Configuration abbreviations for peripheral cannulations for extracorporeal life support. The table shows the whole concept for peripheral cannulation abbreviations. In Panel A, abbreviations for flow cannulae and membrane lung are displayed in a simple and more detailed manner. Panel B shows cannula/catheter tip placement in both simple and more specific ways according to function and size. (PDF 84 kb)

Additional file 2: Examples of the nomenclature used for peripheral, central, and combined cannulation combinations. Examples of the nomenclature used in different cannulation configurations at a variety of level depths. (A) Examples for peripheral cannulation. (B) Examples for central cannulation. (C) Examples for combined cannulation configurations. (PDF 74 kb)

Additional file 3: Rules for the ELSO Maastricht Treaty for ECLS Nomenclature. This file shows a summary of the rules on which the nomenclature for cannulation abbreviation is based. (PDF 40 kb)

## Abbreviations

ECLS: Extracorporeal life support
ECMO: Extracorporeal membrane oxygenation
ELSO: Extracorporeal Life Support Organization
VA: Venoarterial
VV: Venovenous
VVA: Venovenoarterial
