# Supplementary material for: The ELSO Maastricht Treaty for ECLS Nomenclature: abbreviations for cannulation configuration in extracorporeal life support - a position paper of the Extracorporeal Life Support Organization
Source: Crit Care. 2019 Feb 8;23:36. doi: 10.1186/s13054-019-2334-8 (PMC6367794; doi:10.1186/s13054-019-2334-8)
Supplement: Supplementary file 1 — Configuration abbreviations for peripheral cannulations for extracorporeal life support. The table shows the whole concept for peripheral cannulation abbreviations. In Panel A, abbreviations for flow cannulae and membrane lung are displayed in a simple and more detailed manner. Panel B shows cannula/catheter tip placement in both simple and more specific ways according to function and size. (PDF 84 kb) [file 13054_2019_2334_MOESM1_ESM.pdf]

Additional file 1:

Configuration abbreviations for peripheral cannulations for extracorporeal life support[a]

(A) Configuration	Abbreviation	Specifics	Comment
Anatomical primary (X)			
Level one			
Cannula placed in an artery	A		Return of oxygenated blood or drainage for pumpless CO_2 removal
Cannula placed in a vein	V		Drainage or return site
Cannula placed in pulmonary artery	P		Drainage or return of oxygenated blood
Membrane lung	-		
Two parallel membrane lungs	=		
Two serial membrane lungs	+		
No membrane lung in circuit	x		
Dual-lumen cannula (for V-V)	(dl)V-V	(ca)<br>(bc)	(ca) for (dl) if cavo-atrial DLC<br>(bc) for (dl) if bi-caval DLC
Dual-lumen cannula + arterial return	(dl)V-VA	(ca)<br>(bc)	DLC with one arterial reinfusion cannula indexed accordingly (V-VA).
Dual-lumen cannula + arterial return	(dl)VV-A	(ca)<br>(bc)	DLC with Y-piece using both limbs for venous drainage, and one arterial reinfusion cannula.
Dual-lumen cannula with return in the pulmonary artery	(dl)V-P		Venopulmoarterial ECMO supporting the right ventricle. May be equivalent to percutaneous “oxyRVAD”.
Dual-lumen cannula with return in the pulmonary artery	(dl)VxP		Venoarterial ECLS supporting the right ventricle. Note: no ML (x) in circuit, i.e. “peripheral RVAD”.
Pumpless	(pl)A-V		(pl) indicates pumpless driven circuit, A-V
Cephalad draining catheter	c		Drainage of venous blood from the jugular bulb, same side as "V" on neck[b]
Venting catheter	v		Cardiac atrial or ventricular venting catheter[b]
Distal perfusion cannula	a		In V-A for perfusion of cannulated leg via the femoral artery on same side as "A" [b]

	d		For perfusion of cannulated leg via the dorsal foot or posterior tibial artery on same side as "A"[b]
Distal perfusion within the cannula	Aa		Aa underlined indicates dual port femoral arterial cannula (designed with distal port)
Anatomical secondary indexed ***(X_n)***			
Level two			
**(B) Index lettering**	***Simple approach***	***Extended approach***	**Comment**
Level two			
Right atrium	a	a	Right atrium if index for venous cannula (tip position mid to upper part), left atrium if index for venting catheter
Carotid artery	c	cl/cr	Left or right side
Femoral vessel	f	fl/fr<br>flg/frg	Artery or vein, left or right side<br>Chimney graft on left/right femoral artery
Jugular vein	j	jl/jr	Via left or right jugular vein
Subclavian vessel	s	sl/sr<br>slg/srg	Artery or vein, left or right side<br>Chimney graft on left/right subclavian artery
Level three			
Iliacal vessel	i	il/ir	Artery or ven, left or rigth side
Inferor caval vein	ivc	ivc	Venous cannula tip position at level of liver vein
Superior caval vein	svc	svc	Venous cannula tip position in superior vena cava
Venting catheter (indexing for v_x)		v_{al}	Left atrium draining catheter
		v_{ar}	Right atrium draining catheter[b]
		v_c	Left cardiac chamber draining catheter[b]
		v_{ts}	Trans-septal left atrium draining catheter
Level four			
Diameter in French (Fr)	25/….		mm x 3 = Fr (outer cannula diameter)
Cannula length in centimeters	…/18		If diameter not stated, do not print length alone[c]

The A-panel shows abbreviations for flow cannulae and membrane lung in a simple and more detailed manner. The B-panel shows cannula/catheter tip placement both in a simple and in more specific way according to function.

bc, bi-caval design; ca, cavo-atrial dual lumen design; dl, dual lumen; DLC, dual-lumen cannula; ECLS, extracorporeal life support; ECMO, extracorporeal membrane oxygenation; Fr, French; IVC, inferior vena cava; ML, extracorporeal membrane lung; V-A, venoarterial extracorporeal life support; V-V, venovenous extracorporeal life support.

[a]It should be noted that the terms *venous* and *arterial* refer to anatomical structures, not the oxygenation state of the blood.

[b]**a**, **c**, **d,** and **v** are significant draining or reentry points but with lower comparable flows; they are printed as lowercase letters, not indexed.

[c]Differs between manufacturers, may be the insertion length or total cannula length.
