# Supplementary material for: The ELSO Maastricht Treaty for ECLS Nomenclature: abbreviations for cannulation configuration in extracorporeal life support - a position paper of the Extracorporeal Life Support Organization
Source: Crit Care. 2019 Feb 8;23:36. doi: 10.1186/s13054-019-2334-8 (PMC6367794; doi:10.1186/s13054-019-2334-8)
Supplement: Supplementary file 2 — Examples of the nomenclature used for peripheral, central, and combined cannulation combinations. Examples of the nomenclature used in different cannulation configurations at a variety of level depths. (A) Examples for peripheral cannulation. (B) Examples for central cannulation. (C) Examples for combined cannulation configurations. (PDF 74 kb) [file 13054_2019_2334_MOESM2_ESM.pdf]

Additional file 2:

Examples of the Nomenclature used for Peripheral, Central and Combined Cannulation Combinations

The following examples show how the nomenclature system could be used for various configurations with a minimal risk for potential misunderstanding. Additional figures/letters will only provide more information (*Table 2 and Additional file 1*).

A. Examples of peripheral cannulation

- V-V — venovenous configuration.
- V-Ad — venoarterial configuration with a distal perfusion cannula. “d” carries a flow and therefore is not indexed. V-<u>Ad</u> indicates use of an arterial cannula with an *in situ* port for distal perfusion.
- (dl)V_f-V — venovenous configuration that uses a DLC placed via a femoral vein.
- (ca13)V-V — veno-venous configuration with a cavo-atrial design 13Fr DLC.
- (bc19)Vcep-V — 19Fr dual-lumen bi-caval cannula with a cephalad draining catheter (cep is written in lower case, *not indexed*, because it is a genuine draining catheter but with lesser flow). “cep” can only be on the same side as the DLC.
- (dl)VxP — percutaneously placed DLC for atrio-pulmonary artery right ventricular support. Note – no oxygenator in circuit.
- V_f-V_j — venovenous configuration, with femoro-jugular flow direction.
- $V29_{fsvc}$-$V17_{ja}$ — venovenous configuration with a femoral 29 Fr drainage cannula passing through the right atrium with the tip in the superior vena cava for return via a jugular cannula with tip in right atrium.
- P-$A_{fr}d_p$ — draining via cannula in the pulmonary artery for venoarterial ECMO with return via the right femoral artery and distal perfusion applied at the foot.

- $V27/50_{fsvc}$-$A19_{f}$ venoarterial femoro-femoral configuration that uses a 27Fr long drainage cannula placed via a femoral vein with the tip in the SVC and a 19Fr return cannula via a femoral artery. Note that no distal perfusion cannula is reported.

- $V25/38_{ja}$-$A19/18_{f}$d venoarterial configuration, atrio-femoral flow direction through a 25Fr/38 cm cannula placed via the right jugular, tip in upper right atrium for drainage, and a 19Fr/18 cm return cannula via a femoral artery with a distal perfusion cannula.

- $V12_{a}$-$A10_{carr}$ venoarterial configuration atrio-carotid flow direction in an infant using a 12Fr cannula for draining from the right atrium and a 10Fr arterial cannula for return of oxygenated blood via the right carotid artery.

- V_{ivc}-$A_{fl}d_{t}V_{j}$ (V-AV) venoarterial configuration in femoro-femoral flow direction with an additional return cannula of oxygenated blood placed via the right jugular vein. A distal perfusion cannula is used for the leg (left side). V_{ivc}-$\underline{Ad}_{fl}V_{j}$ would be the same configuration but the arterial cannula has the *in situ* port for distal perfusion.

- $Vvnt_{al}$-A the simple form to indicate VA configuration with a left atrial vent drainage catheter.

- $V25/38_{ja}vnt_{al}$-$A19/18_{fr}d_{t}$ indicates the same as the previous configuration but with the added specifics: venous draining cannula 25Fr, 38 cm placed via the right jugular, tip in upper right atrium for drainage, and a venting catheter placed in the left atrium. Further, an arterial 19Fr, 18 cm return cannula and a distal perfusion cannula are placed via the right femoral artery.

B. Examples of central cannulation

- LV-AO central cannulation with the drainage cannula in the left ventricle and the reinfusion cannula in the aorta. The hyphen indicates that an ML is present in conformity to nomenclature for peripheral cannulation.

- RA-AO right atrium drainage via an ML and return to the aorta.

- RV-PA — central cannulation with the drainage cannula in the right ventricle, the reinfusion cannula in the pulmonary artery, and an ML in the circuit.

- RA-PA\LVAO — central dual cannulation: a first assist device with ML is placed with the drainage cannula in the right atrium and reinfusion into the pulmonary artery; a second assist device without ML is placed with the drainage cannula in the left ventricle and the reinfusion cannula in the aorta.

- RVPA\LV-AO — central dual cannulation: a first assist device without ML is placed with the drainage cannula in the right ventricle and reinfusion into the pulmonary artery; a second assist device with ML is placed with the drainage cannula in the left ventricle and the reinfusion cannula in the aorta.

- $RVvnt_{al}$-PA — central cannulation with the drainage cannula in the right ventricle and the reinfusion cannula in the pulmonary artery; a ML is present combined with a left atrial vent.

- plRV-$LACO_2R$ — pumpless central cannulation with the drainage cannula in the right ventricle and the reinfusion cannula in the left atrium for decarboxylation.

- oxyLVAD — left ventricular assist device with ML in circuit for oxygenation.

C. Examples of hybrid cannulation

- V-A/TVLS — V-A ECMO with a transvalvular axial pump device applied (before or during ECMO).

- V_{ja}-/IA — drainage via jugular cannula with tip in right atrium and return to the innominate artery

- V-A/LVAD — V-A ECMO with a left ventricular assist device applied (before or during ECMO).

- (bc31)V_{jr}-V/LVAO — Bi-caval 31Fr DLC via right jugular promoting oxygenation with a concomitant central cannulation, drainage from the left ventricle, and return into the aorta without ML.
- (dl)V-P/RVAD — percutaneously placed DLC for atrio-pulmonary artery V-P ECMO and right ventricular assist device.
- (dl)VxP/oxyLVAD — DLC for percutaneous atrio-pulmonary arterial right ventricular support (before or during ECMO) with an ML in the LVAD circuit for oxygenation.
- PA/V_{fa}-A_f — drainage via one centrally placed cannula in the pulmonary artery and one percutaneous cannula placed via a femoral vein with tip in right atrium. Return is to femoral artery.
- V_{fa}-A_f/PA_{jr} — drainage via femoral vein with the tip in the right atrium and return to the pulmonary and femoral artery.
- V_{fa}-PA/TVLS — Bi-ventricular ECLS with a concomitant venovenous ECMO and right ventricular support (femoral vein cannula in the right atrium, return to the pulmonary artery cannula, and a transvalvular axial pump device applied (before or during ECMO) for left ventricular support.
- V-A/IABP — V-A ECMO with concomitant use of intra-aortic balloon pump.
- V(25/55)$_{fa}$-V(19)$_{jr}$/LVAO — V-V ECMO over a 25Fr/55 cm cannula via unspecified femoral vein (tip in right atrium); the return cannula enters the SVC via the right jugular vein. A central cannulation is applied from the left ventricle to the ascending aorta without ML.

Abbreviations: DLC, dual-lumen cannula; ECMO, extracorporeal membrane oxygenation; IVC, inferior vena cava; ML, extracorporeal membrane lung; LVAD, left ventricular assist device; RVAD, right ventricular assist device; SVC, superior vena cava; TVLS, transvalvular left ventricular support.
