# Supplementary material for: The ELSO Maastricht Treaty for ECLS Nomenclature: abbreviations for cannulation configuration in extracorporeal life support - a position paper of the Extracorporeal Life Support Organization
Source: Crit Care. 2019 Feb 8;23:36. doi: 10.1186/s13054-019-2334-8 (PMC6367794; doi:10.1186/s13054-019-2334-8)
Supplement: Supplementary file 3 — Rules for the ELSO Maastricht Treaty for ECLS Nomenclature. This file shows a summary of the rules on which the nomenclature for cannulation abbreviation is based. (PDF 40 kb) [file 13054_2019_2334_MOESM3_ESM.pdf]

Additional file 3:

The ELSO Maastricht Treaty for ECLS Nomenclature:

Rules for the use of the Abbreviations for Cannulation Configuration in Extracorporeal Life Support (ECLS)

The Nomenclature covers all modalities of ECLS and is divided into four levels. Every level has the whole alphabet to use for denomination of a specific entity. Central and peripheral cannulation configured ECLS and long-term life support devices used in combination or independently in parallel are all included in this nomenclature.

All abbreviations are read in the anticipated blood flow direction, i.e. from left to right.

An abbreviation formula never contains spaces/blanks.

The interface between drainage and return sides is denominated by the position of the membrane lung (-, =, +, x). A slash (/) denotes the separation when a combination of peripheral and central configurations is used or marks the separation of cannula diameter and length in level four.

Two independent devices used independently during the same time is separated using a back slash (\).

The levels two, three and four may be used in part and independently of the other ones. The ECLS provider may adopt to, and use the nomenclature according to own goals and level of depth, e.g. quality assurance, research and development etc.

Level one:

Uppercase letters are used for major flow cannulae. For peripheral cannulation one uppercase letter is used. For central cannulation the major flow cannula is expressed with two uppercase letters. A ventricular support device is abbreviated as is (to exemplify: VLAD).

Lowercase letters are used for supportive (minor) flow cannulae in both peripheral and central cannulation configurations.

In the abbreviation, the minor flow cannula is placed directly after the major flow cannula to which it supports.

Lowercase letters within parenthesis are used to indicate a pumpless (pl) driven circuit as in, or to specify a dual-lumen (dl) cannula (DLC). The specification is always placed before the first major flow letter of the abbreviation, for a pumpless circuit (pl)A-V, and for DLC, (dl)V-V.

If cannula configuration is changed by placement of additional cannula/cannulae, the initial configuration maintains as the core. Additional drainage cannula will be positioned outside to (left)

the pre-existing letter/s on the drainage side. An additional return cannula will be placed to the outside (right) of the pre-existing return cannula letter/s. This may transform a VV or VA mode into the hybrid-mode VVA. The nomenclature thus contains inherent information of chronology of the ECLS run. In case of implementation of a hybrid-configuration from start the provider may put the most important mode for that patient as the core configuration. However, in terms of physiology and support given this is of less importance.

Level two and three:

Indexed/subscript letters are used to increase information for any major or minor flow cannula.

In communication systems not permitting indexing/subscript of letters, parenthesis should be used to mark the hierarchy of those letters.

Indexed letter/s follows directly after the letter for the major (uppercase) or minor (lowercase) cannula it specifies.

If a specific minor flow cannula to its function only can be a conjunct to a specific major flow cannula, such minor flow cannula should not be specified with additional index letters. In certain cases, additional index letter/s may be used that specific information is important for the user of the nomenclature. Letters used are limited to the contemporary definition and denomination for the Nomenclature.

The order of indexed letters in each specification are:

Level two

1) Cannulation site, i.e. the punctured vessel
2) Cannulation side, left (l) or right (r)

Level three

3) Cannula tip position
4) Side may be given in case cannulation site (level two) not is used, left (l) or right (r)
5) Other, e.g. chimney graft (g)

Level four:

Cannula dimension is noted directly after cannula letter/s but before any additional subscripts for that cannula, ($V25_{fli}$, $V17/18_{j}$). The cannula diameter by the manufacturers in French units (1 Fr = 1/3 mm outer diameter) should be used, (V25-A, V17-A15). The cannula length in centimeters (by manufacturer) may be added after the respective cannula dimeter with a slash (/) to separate, (V25/38-A, V17/18-A15/17). Length should only be added if diameter is reported.
Note: There is currently no consensus for definition of cannula length among manufacturers.
